# Atomoxetine treatment and ADHD-related difficulties as assessed by adolescent patients, their parents and physicians

**DOI:** 10.1186/1753-2000-3-21

**Published:** 2009-08-24

**Authors:** Ralf W Dittmann, Peter M Wehmeier, Alexander Schacht, Anette Minarzyk, Martin Lehmann, Kathrin Sevecke, Gerd Lehmkuhl

**Affiliations:** 1 Department of Child and Adolescent Psychiatry, Central Institute of Mental Health Mannheim, University of Heidelberg, Germany; 2 Lilly Deutschland GmbH, Medical Department, Bad Homburg, Germany; 3 Department of Child and Adolescent Psychiatry, University of Cologne, Germany

## Abstract

**Background:**

The degree of ADHD-related difficulties – reflecting overall impairment, social functioning, and quality of life – may be perceived differently by adolescent patients, parents and physicians. The primary aim of this study was to investigate ADHD-related difficulties during atomoxetine treatment, as perceived by the three different raters. Secondary objectives focused on effectiveness and tolerability of atomoxetine treatment in a population of adolescent patients with ADHD.

**Methods:**

Adolescents with ADHD, aged 12–17 years, received open-label atomoxetine (0.5–1.2 mg/kg/day) up to 24 weeks. ADHD-related difficulties at various times of the day were rated using the Global Impression of Perceived Difficulties (GIPD) instrument. Inter-rater agreement was analyzed using Cohen's Kappa with 95% confidence intervals (95% CI). ADHD-Rating Scale (ADHD-RS) and Clinical Global Impression Severity (GGI-S) scores were assessed by the investigator; and spontaneous adverse events, vital signs and laboratory parameters were collected for tolerability assessments.

**Results:**

159 patients received atomoxetine. Patients' baseline mean GIPD total ratings were significantly lower than parents' and physicians' scores (12.5 [95%CI 11.6;13.5] vs. 17.2 [16.2;18.2] and 18.8 [17.8;19.8]). For all raters, GIPD scores significantly improved over time. Changes were greatest within the first two weeks. Kappa coefficients varied between 0.186 [0.112;0.259] and 0.662 [0.529;0.795], with strongest agreements between parent and physician assessments, and significant improvements of patient/physician agreements over time (based on 95% CIs). ADHD-RS and CGI-S scores significantly improved over the course of the study (based on 95% CIs). Tolerability results were consistent with earlier reports.

**Conclusion:**

ADHD-related difficulties were perceived differently by the raters in this open-label trial, but consistently improved during atomoxetine treatment. The GIPD instrument appeared sensitive to treatment-related change. These primarily quantitative findings may guide future studies to more systematically investigate the clinical and practical relevance of the differences observed. Additionally, in order to further validate these results, placebo- and comparator-controlled trials are recommended as well as inclusion of healthy controls and other patient populations.

**Trial Registration:**

**Clinical Trial Registry**: ClinicalTrials.gov: NCT00191737

## Background

Attention-deficit/hyperactivity disorder (ADHD) is characterized by inattention, impulsivity, and hyperactivity and affects 3–7% of school-aged children in the United States [1]. ADHD is associated with significant impairment of cognitive and psychosocial functioning [2,3] and quality of life (QoL) in patients and families [4-7].

Beyond the improvement of core symptoms during ADHD treatment, there is growing appreciation of possible additional patient and family benefits, including QoL and functional outcome parameters [8-10].

Atomoxetine is a non-stimulant treatment option for ADHD [11,12], efficacy and tolerability in children and adolescents have been demonstrated in a number of placebo-controlled randomized clinical trials [8,10,13-16], supported by various meta-analyses [17-20]. These studies have primarily used investigator-rated questionnaires such as the ADHD Rating Scale (ADHD-RS) [21,22] and the Clinical Global Impression (CGI) [23,24] as outcome measures for the core symptoms of ADHD. Other questionnaires, such as the Child Health Questionnaire (CHQ) [25] and Child Health and Illness Profile – Child Edition (CHIP-CE) [26] assess aspects of ADHD that go beyond the core symptoms of the disorder and reflect various dimensions of health-related QoL. To date, several studies have shown improvement in health-related QoL of children and adolescents treated with atomoxetine [8,9,27-31].

However, when assessing QoL in adolescents with ADHD, both, symptom severity and ADHD-related difficulties, may be perceived and rated differently by patients, parents, and physicians [7]. For example, children and adolescents may perceive and rate the difficulties associated with ADHD as less severe than their parents do [32]. Health-related QoL is a multidimensional concept that reflects the subjective physical, social and psychological aspects of health and is distinct from symptoms of the disorder and objective functional outcomes [33]. QoL closely depends on the subjectively perceived impact of the disorder (and of the respective treatment) on the level of physical, psychological and social functioning [34,35]. The severity of difficulties related to the disorder as perceived by the patients may therefore be considered a good indicator reflecting QoL, beyond the symptoms assessed on the various scales based on the diagnostic items from the DSM-IV [e.g., [21,22]] This patient perspective may be compared with the perspectives of parents and physicians. Also, symptoms (e.g., inattention) of the disorder itself may contribute to altered perceptions of those difficulties by the patient.

Thus, the **primary objective **of this study was to investigate the severity of ADHD-related difficulties perceived by patients, parents, and physicians during atomoxetine treatment, and to compare these three perspectives over the course of the study, using the Global Impression of Perceived Difficulties (GIPD) instrument. The psychometric properties of this brief scale have recently been reported [36]; it has especially been devised to capture the perception of the patient's ADHD-related difficulties from a patient, parent, and physician perspective (with adjusted wording for each). The GIPD instrument can be considered to reflect overall impairment, psychosocial functioning, and quality of life (QoL); sensitivity to treatment-related change over time has also been indicated [37]. It consists of five items which assess ADHD-related difficulties at the typical situations over the course of the day when ADHD patients face their main problems. Each item is rated on a seven-point scale.

As German ethic committees tend to be rather reluctant towards placebo-controlled studies in juvenile patients, and efficacy was not a main objective in this post-launch study, we decided on a single-arm, open-label design for reasons of feasibility.

**Secondary objectives **were to evaluate the effectiveness and tolerability of atomoxetine in adolescents with ADHD. Results from a parallel study performed in children with ADHD (6 – 11 years of age) have been published [37].

## Methods

### Study design and procedures

This multicenter, open-label, single-arm study was designed to investigate the degree of ADHD-related difficulties in adolescents with ADHD treated with atomoxetine as perceived by patients, parents, and physicians. Patients were recruited at 35 child and adolescent psychiatry and pediatric practices and outpatient clinics throughout Germany. Boys and girls aged 12–17 years with ADHD as defined in DSM-IV-TR were eligible for the study. The diagnosis was confirmed using the "Diagnose-Checkliste Hyperkinetische Störungen" (Diagnostic Checklist for Hyperkinetic Disorders), a structured standard instrument based on the respective DSM-IV-TR and ICD-10 criteria [38,39] which is routinely used for diagnostic assessment of ADHD in Germany. Comorbid psychiatric and somatic disorders were assessed as part of a careful clinical examination performed by the investigators (board-certified child and adolescent psychiatrists or pediatricians).

Patients were to have an IQ of ≥70 based on the clinical judgment of the investigator. The exclusion criteria comprised significantly abnormal laboratory findings, acute or unstable medical conditions, cardiovascular disorder, history of seizures, pervasive developmental disorder, psychosis, bipolar disorder, suicidal ideation, any medical condition that might increase sympathetic nervous system activity, or the need for psychotropic medication other than study drug. Patients already being treated with atomoxetine were also excluded. The protocol was approved by the ethics committee of the University of Cologne, Germany, and the study was conducted in accordance with the principles of the Declaration of Helsinki.

Following a wash-out period, baseline assessments were carried out with all the instruments used. During the first week, the patients were treated with atomoxetine at a dose of approximately 0.5 mg/kg per day. During the following 7 weeks, the recommended atomoxetine dose was 1.2 mg/kg per day, which could be adjusted within a range of 0.5 – 1.4 mg/kg per day, depending on effectiveness and tolerability. Medication was given once-a-day in the morning. Assessments were carried out weekly during the first two weeks of treatment, and every two weeks thereafter. After the 8-week treatment period, the physicians decided in accordance with the patients and their parents whether the patient was going to continue treatment for further 16 weeks, considering both effectiveness and tolerability/safety of the compound for the respective patient. Patients who participated in this extension period continued on the same atomoxetine dose which again could be adjusted within a range of 0.5 – 1.4 mg/kg per day if necessary. During the extension period, three assessments were carried out at 12, 16, and 24 weeks after baseline.

The Global Impression of Perceived Difficulties (GIPD) instrument was used as the primary outcome measure. The GIPD is a validated instrument [36] that has especially been developed to capture the perception of the patient's ADHD-related difficulties from a patient, parent (or primary caregiver), and physician perspective, and can be considered to reflect overall impairment, psychosocial functioning, and quality of life (QoL) [37]. The GIPD consists of five items which assess ADHD-related difficulties at the typical times of the day, when ADHD patients face their main problems: (1) in the morning, (2) during school, (3) during homework, (4) in the evening, and (5) overall difficulties over the entire day and night. Each item is rated on a seven-point scale (1 = not at all difficult, 7 = extremely difficult) in analogy to the CGI-Severity scoring [23,24], and reflects the situation during the previous week. There are three different versions with adjusted wording for each rater, allowing comparisons. The GIPD Total score was calculated for each rater group by summation of item scores (range 5 to 35). If one item was missing, the total score was also considered as missing. The Attention-Deficit/Hyperactivity Disorder Rating Scale-IV-Parent Version: Investigator-Administered and Scored (ADHD-RS-IV-Parent:Inv) is an 18-item scale, with one item for each of the 18 ADHD symptoms listed in DSM-IV-TR [21,22]. There are 2 subscales: the "hyperactivity/impulsivity" subscale is the sum of the even items, and the "inattention" subscale is the sum of the odd items. This scale is scored by an investigator while interviewing the parent or primary caregiver.

The Clinical Global Impression-Severity-Attention-Deficit/Hyperactivity Disorder Scale (CGI-S-ADHD) is a seven point single-item rating scale of the physician's assessment of the severity of ADHD symptoms [23,24].

Following the clinical interview with patients and parents, and the completion of ADHD-RS-IV and CGI-S-ADHD scales by the investigator, GIPD ratings were done independently by patients and parents during each office visit. The investigator would then score the GIPD physician version taking into account the patient and parent GIPD scorings from the respective visit plus all additional information about the patient provided to him. Adverse event assessment concluded the session. Thus, GIPD ratings from patients and parents were not used to inform ADHD-RS or CGI-S ratings by the investigator or to guide treatment decisions, e.g., dose-adjustments. Given the open-label design of the study, this sequence was also chosen to resemble the routine (naturalistic) course of an office visit.

### Sample size and statistical analysis

For calculating an appropriate sample size, we assumed that the true value of Kappa [40] for the GIPD scale is 0.8 (between patients and parents as well as between patients and physicians). The respective two-sided 95% confidence intervals were intended to extend 0.1 from the observed value of Kappa for the estimate to be sufficiently precise. Furthermore, we assumed a true response rate of 50%. Thus, a sample size of 139 patients was considered sufficient for the desired precision. Assuming a proportion of 5% of patients with unspecified data on the GIPD scale, a sample size of 147 patients was planned.

The data of all patients were evaluated (Full Analysis Set, FAS). The dataset for all analyses of changes from baseline to endpoint consisted of all patients with a baseline measurement and at least one post-baseline measurement during the 8-week treatment period.

In addition to Last Observation Carried Forward (LOCF) analyses and Observed Cases (OC) analyses, LOCF-BR (LOCF – baseline rater) and OC-BR (OC – baseline rater) analyses were applied: Values not rated by the same individual both at baseline and later on (e. g., father rated at baseline, mother rated later) were replaced by the last value from the baseline rater (if present), otherwise the value was deleted. Obviously, this applied only to the parent rating scales.

Evaluation was largely descriptive. All tests of statistical significance were carried out at a nominal level of 5% using two-tailed test procedures. Two-sided confidence intervals (CIs) were computed using a 95% confidence level. Cohen's weighted Kappa (Kappa) with 95% confidence intervals [40] was used to determine the agreement between patients and parents, patients and physicians, and between parents and physicians. Kappa-calculations were based on OC values and OC-BR for parents, respectively. All other tables and scores cited in the text represent LOCF values (LOCF-BR for GIPD parent ratings, respectively) whilst the figures are based on OC values. Subgroup analyses were performed for ADHD-subtypes (DSM-IV-TR criteria).

## Results

### Patient population and disposition

**Figure 1:**
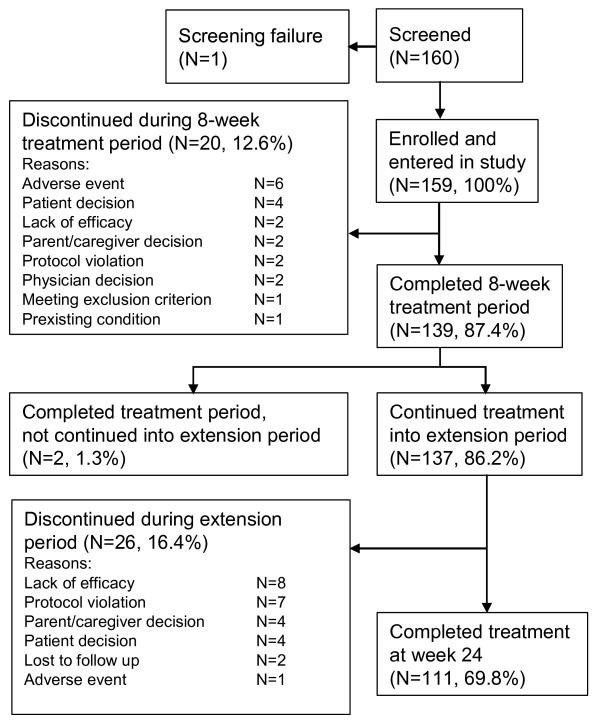
**Patient disposition**.

Of the 160 patients screened, 159 patients (100%) were enrolled in the study and treated with atomoxetine, 137 (86.2%) patients completed the treatment period and continued into the extension period. 20 (12.6%) patients discontinued early over the course of the 8 week treatment period, two more (1.3%) completed the treatment period, but did not continue into the extension period based on the decision of the physician. 26 (16.4%) patients discontinued between weeks 8 and 24. 111 (69.8%) patients completed the study at week 24. Discontinuations were mostly due to lack of efficacy. All reasons for discontinuation are shown in Figure 1.

Table 1 shows the patient characteristics. Male patients and those with the combined subtype of ADHD appeared to be younger and to be diagnosed earlier than girls or patients with the predominantly inattentive subtype. 68 (54.4%) boys and 13 (38.2%) girls were diagnosed with the combined subtype. The predominantly inattentive subtype was diagnosed in 53 (42.4%) boys and 20 (58.8%) girls.

**Table 1:** Patient characteristics

	**N**	**%**	**Age (years)**		**Height (cm)**		**Weight (kg)**		**Age at start of symptoms (years)**		**Age at ADHD diagnosis (years)**	
	
			**Mean**	**SD**	**Mean**	**SD**	**Mean**	**SD**	**Mean**	**SD**	**Mean**	**SD**
**All patients**	159	100	14.1	1.53	163	10.96	54.4	13.40	4.3	2.21	9.8	2.77
Girls	34	21.4	14.4	1.70	162	8.27	55.3	12.96	4.9	2.50	10.7	2.91
Boys	125	78.6	14.0	1.48	163	11.60	54.2	13.56	4.2	2.11	9.6	2.69
ADHD, combined type*	81	50.9	13.9	1.50	161	11.09	53.8	14.41	4.1	2.13	9.5	2.68
ADHD, predominantly inattentive type*	73	45.9	14.2	1.53	165	10.85	54.3	11.90	4.5	2.03	10.1	2.87
ADHD, NOS*	5	3.1	15.0	1.80	167	4.51	67.2	13.19	6.3	4.58	11.2	2.51

* According to Diagnostic and Statistical Manual of Mental Disorders, Fourth Edition™.

SD = Standard Deviation, NOS = not otherwise specified

Consisting of only 5 subjects, the subgroup of patients with ADHD, not otherwise specified (NOS) was too small for further detailed subgroup analyses. There were no patients meeting criteria for the predominantly hyperactive-impulsive subtype. For the entire patient sample, the mean time span between first occurrence of symptoms (parent report) and first professional diagnosis amounted to approximately 5.5 years.

137 (86.2%) of the 159 adolescents had previously been treated for ADHD. The percentages of pretreated patients differed slightly with respect to sex (boys: N = 109, 87.2%, girls: N = 28, 82.4%) or ADHD-subtype (combined subtype N = 67, 82.7%, predominantly inattentive subtype N = 65, 89.0%). Most frequently used compounds had been short-acting methylphenidate (N = 119, 74.8%), long-acting methylphenidate (N = 92, 57.9%), amphetamines (N = 17, 10.7%), and antidepressants (N = 7, 4.4%). Commonly reported non-drug therapies prior to study start were: structured psychotherapy (N = 22, 13.8%), occupational therapy (N = 11, 6.9%), and other forms of psychotherapy (N = 10, 6.3%). The most frequent reason for discontinuation of any previous therapy was inadequate response (N = 88, 64.2%).

At baseline, patients (N = 155–158) were rated with the following mean scores (± SD): GIPD Total: patient 12.5 (± 5.8), parent 17.2 (± 6.3), physician 18.8 (± 6.0); ADHD-RS-IV: 28.4 (± 10.1), and CGI-S-ADHD: 4.8 (± 0.9).

The mean atomoxetine dose (± SD) given during the first week of treatment was 0.51 (± 0.06) mg/kg per day (minimum 0.40, maximum 0.60 mg/kg per day). Thereafter, the mean dose ranged between 1.17 and 1.19 mg/kg per day (minimum 0.40, maximum 1.40 mg/kg per day). With respect to ADHD-subtype or sex, mean doses were largely within the same range. Overall compliance was at least 96.5% over the entire course of the study according to investigator assessment.

Concomitant medication was taken by 99 (62.3%) of the patients. Analgesics (N = 37, 23.3%), cough and cold remedies (N = 22, 13.8%), antibiotics (N = 18, 11.3%), phytotherapeutics (N = 14, 8.8%; herbal remedies to treat common colds and upper respiratory tract infections), and medications for gastrointestinal diseases (N = 7, 4.4%) were reported most frequently. Continuous behaviour therapy (ongoing before study start) was applied in 10 (N = 6.3%) patients, and 2 (1.3%) patients received occupational therapy. Pre-existing concomitant conditions were reported for 105 (66.0%) patients, the most frequent being psychiatric comorbidities, i.e., conduct disorder (N = 29, 18.2%), oppositional defiant disorder (N = 21, 13.2%), emotional disorder of childhood (N = 4, 2.5%). Two patients had depressed mood (N = 2, 1.3%), none had concomitant anxiety disorder. Physical comorbidities reported at a rate of >2% were headache (N = 10, 6.3%), seasonal allergy (N = 9, 5.7%), acne (N = 8, 5.0%), and asthma and atopic dermatitis (N = 7, 4.4% each).

### GIPD: course over time and agreement between perspectives

**Figure 2:**
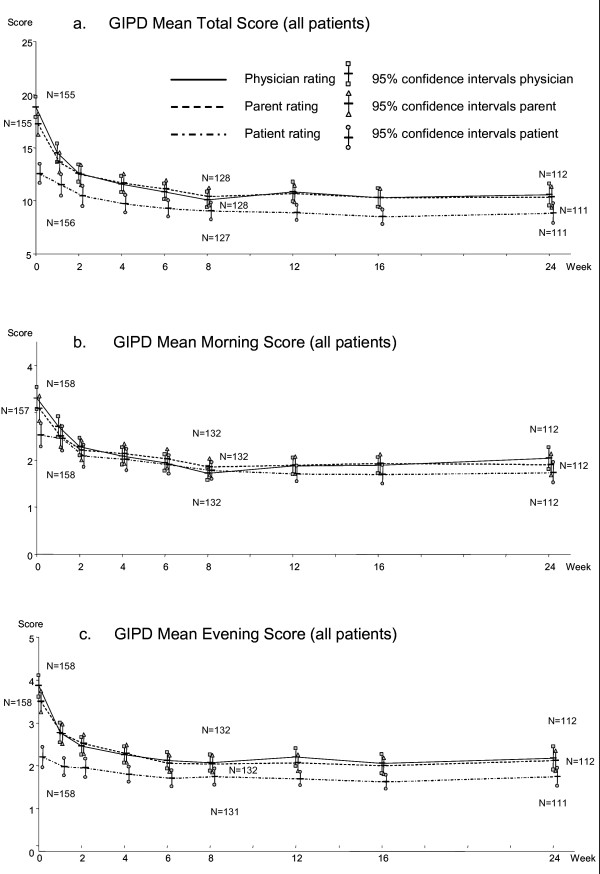
**GIPD total (a), morning (b) and evening (c) scores, as rated by patients, parents and physicians (OC analysis)**.

The mean GIPD total scores for the three rater groups (patients, parents and physicians) took parallel courses over time (Figure 2a). At baseline, parents rated the ADHD-related difficulties somewhat less severe than physicians (not significant; n. s.), but the parent and physician mean total scores converged as early as week 2, and overlapped for the remainder of the study (Table 2). Compared to the parent and physician ratings, the adolescents perceived their difficulties as significantly less severe at most time points throughout the study, (cf. 95% CIs). Mean GIPD total scores improved significantly for all three rater groups from baseline to week 8 and week 24 (cf. 95% CIs).

**Table 2:** Global Impression of Perceived Difficulties (GIPD) total scores, rated by patients, parents and physicians, by ADHD subtype

	**GIPD total score**								
	**Patient rated**			**Parent rated**			**Physician rated**		
	
**ADHD subtype**	**N**	**Mean**	**95% CI**	**N**	**Mean**	**95% CI**	**N**	**Mean**	**95% CI**
**All**									
Week 0	156	12.5	11.6 – 13.5	155	17.2	16.2 – 18.2	155	18.8	17.8 – 19.8
Week 2	157	10.5	9.6 – 11.4	148	12.6	11.7 – 13.6	158	12.6	11.8 – 13.5
Week 8	158	9.6	8.7 – 10.5	150	11.4	10.5 – 12.4	158	11.0	10.2 – 11.9
Week 24	158	9.9	8.9 – 10.9	153	11.7	10.7 – 12.7	158	12.1	11.0 – 13.1

**Combined type**									
Week 0	79	12.6	11.4 – 13.8	79	17.8	16.4 – 19.2	79	19.6	18.3 – 21.0
Week 2	80	10.7	9.4 – 11.9	76	13.4	12.0 – 14.8	81	13.8	12.5 – 15.0
Week 8	81	10.1	8.9 – 11.3	77	11.9	10.7 – 13.2	81	12.4	11.1 – 13.7
Week 24	81	10.0	8.7 – 11.3	77	11.9	10.6 – 13.2	81	12.9	11.4 – 14.3

**Predominantly inattentive type**									
Week 0	72	12.5	11.0 – 14.0	71	16.4	14.9 – 17.9	71	17.9	16.5 – 19.3
Week 2	72	10.1	8.8 – 11.5	67	11.7	10.4 – 13.0	72	11.3	10.3 – 12.3
Week 8	72	9.0	7.7 – 10.3	68	10.7	9.4 – 12.1	72	9.6	8.5 – 10.7
Week 24	72	9.6	8.1 – 11.1	71	11.4	9.9 – 12.8	72	11.2	9.7 – 12.6

Patient and physician ratings based on LOCF (Last Observation Carried Forward), parient ratings are for LOCF-BR (LOCF, Baseline Rater:

Values not rated by the same individual both at baseline and later on (e. g., father rated at baseline, mother rated later) were replaced

by the last value from the baseline rater (if present), otherwise the value was deleted.

CI = Confidence Interval

With respect to GIPD total scores for different ADHD subtypes (Table 2), parents and physicians at baseline rated the ADHD-related difficulties of the adolescents with combined subtype as slightly more severe than the difficulties of the predominantly inattentive subtype (n. s.). In contrast, there was no difference between the patient ratings of the two ADHD-subtypes.

Table 3 and 4 summarize the course of GIPD subscores for morning and evening behaviour (Items 1 and 4 of GIPD total score). Mean values and course over time (Figure 2) were similar as for the GIPD total score. All respective differences between rater groups and ADHD subtypes were found in the evening scores as well. Regarding the GIPD morning score, however, patient-rated mean scores over time were found close to those for parent and physician ratings after baseline. The patients' evening rating was significantly lower than the parents' and physicians' evening rating between baseline and week 2. Comparing the mean GIPD morning and evening scores, it was found that patients generally tended to rate their difficulties in the evening lower than those in the morning, whereas parents and physicians tended to perceive evening difficulties as more severe (physicians significantly at baseline).

**Table 3:** GIPD morning scores, rated by patients, parents and physicians, by ADHD subtype

	**GIPD morning score**								
	**Patient rated**			**Parent rated**			**Physician rated**		
	
**ADHD subtype**	**N**	**Mean**	**95% CI**	**N**	**Mean**	**95% CI**	**N**	**Mean**	**95% CI**
**All**									
Week 0	158	2.5	2.3 – 2.8	157	3.1	2.8 – 3.4	158	3.3	3.1 – 3.5
Week 2	157	2.1	1.9 – 2.3	148	2.2	2.0 – 2.5	158	2.3	2.1 – 2.5
Week 8	158	2.0	1.8 – 2.2	150	2.1	1.9 – 2.3	158	1.9	1.7 – 2.1
Week 24	158	1.9	1.7 – 2.2	153	2.1	1.9 – 2.4	158	2.2	2.0 – 2.4

**Combined type**									
Week 0	80	2.6	2.2 – 2.9	80	3.2	2.8 – 3.6	80	3.4	3.1 – 3.7
Week 2	80	2.2	1.8 – 2.6	76	2.4	2.1 – 2.7	81	2.5	2.2 – 2.7
Week 8	81	2.1	1.8 – 2.4	77	2.2	1.9 – 2.5	81	2.2	1.9 – 2.5
Week 24	81	2.0	1.7 – 2.4	77	2.2	1.9 – 2.5	81	2.4	2.1 – 2.7

**Predominantly inattentive type**									
Week 0	73	2.5	2.1 – 2.8	72	3.0	2.6 – 3.4	73	3.2	2.9 – 3.6
Week 2	72	2.0	1.7 – 2.3	67	2.1	1.8 – 2.3	72	2.1	1.8 – 2.3
Week 8	72	1.8	1.5 – 2.1	68	2.0	1.7 – 2.3	72	1.7	1.4 – 1.9
Week 24	72	1.8	1.5 – 2.1	71	2.1	1.7 – 2.4	72	2.0	1.7 – 2.3

Patient and physician ratings based on LOCF (Last Observation Carried Forward), parent ratings are for LOCF-BR (LOCF, Baseline Rater:

Values not rated by the same individual both at baseline and later on (e. g., father rated at baseline, mother rated later) were replaced by the last value from the baseline rater (if present), otherwise the value was deleted.

CI = Confidence Interval

**Table 4:** GIPD evening scores, rated by patients, parents and physicians, by ADHD subtype

	**GIPD evening score**								
	**Patient rated**			**Parent rated**			**Physician-rated**		
	
**ADHD subtype**	**N**	**Mean**	**95% CI**	**N**	**Mean**	**95% CI**	**N**	**Mean**	**95% CI**
**All**									
Week 0	158	2.2	2.0 – 2.4	158	3.5	3.3 – 3.7	158	3.9	3.6 – 4.1
Week 2	158	1.9	1.7 – 2.1	149	2.6	2.3 – 2.8	158	2.5	2.3 – 2.7
Week 8	158	1.8	1.6 – 2.0	150	2.2	2.0 – 2.4	158	2.2	2.0 – 2.4
Week 24	158	1.9	1.7 – 2.1	153	2.3	2.1 – 2.6	158	2.4	2.2 – 2.7

**Combined type**									
Week 0	80	2.4	2.0 – 2.7	80	3.7	3.4 – 4.1	80	4.2	3.8 – 4.5
Week 2	81	2.1	1.8 – 2.4	77	2.9	2.5 – 3.2	81	2.8	2.5 – 3.1
Week 8	81	1.9	1.7 – 2.2	77	2.4	2.1 – 2.7	81	2.5	2.2 – 2.9
Week 24	81	1.9	1.6 – 2.2	77	2.4	2.0 – 2.7	81	2.6	2.3 – 3.0

**Predominantly inattentive type**									
Week 0	73	2.1	1.7 – 2.4	73	3.3	2.9 – 3.6	73	3.5	3.2 – 3.9
Week 2	72	1.8	1.5 – 2.0	67	2.3	1.9 – 2.6	72	2.1	1.9 – 2.4
Week 8	72	1.7	1.4 – 2.0	68	2.1	1.8 – 2.4	72	1.9	1.6 – 2.1
Week 24	72	1.8	1.5 – 2.1	71	2.4	2.1 – 2.7	72	2.2	1.9 – 2.6

Patient and physician ratings based on LOCF (Last Observation Carried Forward), parent ratings are for LOCF-BR (LOCF, Baseline Rater:

Values not rated by the same individual both at baseline and later on (e. g., father rated at baseline, mother rated later) were replaced by the last value from the baseline rater (if present), otherwise the value was deleted.

CI = Confidence Interval

The calculation of Cohen's Kappa coefficients for the GIPD total score (all patients) revealed an overall increase of agreement between the three rater groups over the course of the study. This improvement was statistically significant for the agreement between patients and physicians (cf. 95% CIs, Table 5).

**Table 5:** Agreement (Cohen's Kappa coefficients) between patient-, parent- and physician rated GIPD total scores, by ADHD subtype

	**Agreement between**								
	**Physician and parent**			**Patient and parent**			**Patient and physician**		
	
**ADHD subtype**	**N**	**Kappa**	**95% CI**	**N**	**Kappa**	**95% CI**	**N**	**Kappa**	**95% CI**
**All**									
Week 0	153	0.533	0.451 – 0.615	153	0.221	0.132 – 0.310	154	0.186	0.112 – 0.259
Week 2	128	0.550	0.460 – 0.641	127	0.359	0.257 – 0.461	147	0.391	0.294 – 0.489
Week 8	112	0.538	0.443 – 0.633	111	0.318	0.205 – 0.432	126	0.385	0.284 – 0.485
Week 24	104	0.639	0.552 – 0.725	103	0.363	0.255 – 0.471	111	0.425	0.319 – 0.532

**Combined type**									
Week 0	79	0.504	0.382 – 0.626	78	0.155	0.041 – 0.270	78	0.131	0.045 – 0.216
Week 2	68	0.509	0.375 – 0.644	67	0.313	0.184 – 0.442	74	0.411	0.287 – 0.534
Week 8	61	0.454	0.306 – 0.602	62	0.222	0.081 – 0.362	66	0.347	0.209 – 0.484
Week 24	55	0.662	0.529 – 0.795	55	0.382	0.237 – 0.527	57	0.402	0.267 – 0.536

**Predom. inattentive type**									
Week 0	69	0.551	0.439 – 0.663	70	0.280	0.142 – 0.419	71	0.237	0.113 – 0.360
Week 2	55	0.556	0.437 – 0.674	55	0.399	0.235 – 0.562	68	0.339	0.190 – 0.489
Week 8	48	0.631	0.511 – 0.751	46	0.434	0.250 – 0.619	56	0.419	0.270 – 0.568
Week 24	45	0.609	0.499 – 0.720	44	0.320	0.159 – 0.481	50	0.438	0.263 – 0.613

Patient and physician ratings based on LOCF (Last Observation Carried Forward), parent ratings are for LOCF-BR (LOCF, Baseline Rater: Values not rated by the same individual both at baseline and later on (e. g., father rated at baseline, mother rated later) were replaced by the last value from the baseline rater (if present), otherwise the value was deleted.

CI = Confidence Interval

The highest degree of agreement was found between physicians and parents. Agreement between patients and parents as well as agreement between patients and physicians were both markedly below the agreement between physicians and parents, with differences in Kappa coefficients reaching statistical significance at various points in time throughout the study (cf. 95% CIs).

Largely the same pattern as for the entire sample was observed in patients with the ADHD combined type, while patients with the predominantly inattentive ADHD-subtype displayed a slightly higher degree of agreement with their parents and physicians (n. s.).

### ADHD Rating Scale (ADHD-RS)

**Figure 3:**
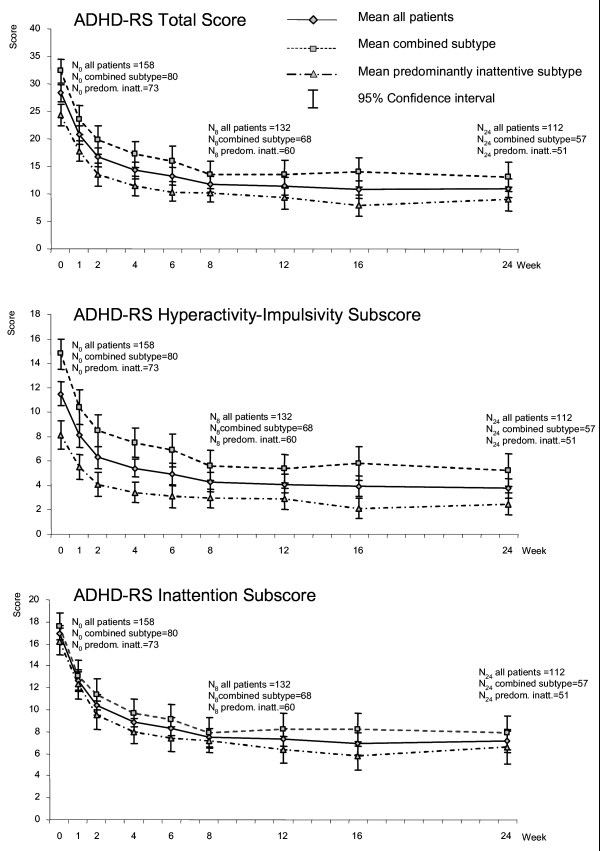
**ADHD Rating Scale (ADHD-RS-Par:Inv), a) total score, b) hyperactivity-impulsivity subscore, c) inattention subscore (for all patients and by ADHD subtype, OC analysis)**.

During the first two weeks of atomoxetine treatment, mean total scores for the investigator-rated ADHD-RS (ADHD-RS-IV-Parent:Inv) significantly decreased from 28.4 [26.8 to 29.9] at baseline to 16.7 [15.0 to 18.3] at week 2 (mean [95% CI]; LOCF). Total scores were at 12.9 [11.4 to 14.4] by week 8, and at 13.3 [11.7 to 15.0] by the end of week 24. The course was largely parallel for both ADHD subtypes (Figure 3a). Over the entire time period, patients of the combined subtype had significantly higher scores than patients of the predominantly inattentive subtype (combined subtype, baseline: 32.4 [30.2 to 34.5], week 2: 19.8 [17.3 to 22.3], week 8: 15.4 [13.0 to 17.9], week 24: 15.7 [13.2 to 18.2], predominantly inattentive subtype, baseline: 24.3 [22.4 to 26.3], week 2: 13.4 [11.4 to 15.4], week 8: 10.3 [8.8 to 11.8], week 24: 11.1 [9.1 to 13.1]).

Looking at the ADHD-RS sub-scores, courses of the Hyperactivity-Impulsivity and Inattention sub-scores followed the general pattern shown for the total score. Combined subtype of ADHD was again associated with statistically significantly higher mean scores than the predominantly inattentive subtype, this held for the Hyperactivity-Impulsivity subscore but not the Inattention subscore (Figures 3b and 3c).

### Clinical Global Impression (CGI-S)

**Figure 4:**
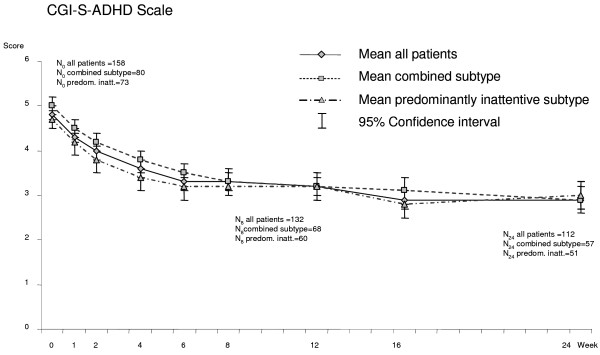
**Clinical Global Impression – Severity (CGI-S-ADHD), for all patients and by ADHD subtype (OC analysis)**.

The mean CGI-S-ADHD score (LOCF) for the overall sample significantly decreased from 4.8 [95%CI 4.7 to 5.0] at baseline to 3.4 [3.2 to 3.6] at week 8 and stayed stable thereafter until week 24 (3.3 [3.1 to 3.5]). Regarding the ADHD-subtypes, a comparable decrease was observed. The mean CGI-S-ADHD scores of the predominantly inattentive subtype tended to be slightly lower than the scores of the combined type over the entire course of the study, but the differences did not reach statistical significance (Figure 4).

### Tolerability

Investigators reported treatment emergent adverse events in 124 (78.0%) patients over the entire study period. Adverse events reported in more than 5% of all patients (N = 159) were: fatigue 45 (28.3%), headache 38 (23.9%), nausea 30 (18.9%), nasopharyngitis 21 (13.2%), vomiting 21 (13.2%), upper abdominal pain 12 (7.5%), decreased appetite 12 (7.5%), dizziness 12 (7.5%), diarrhea 9 (5.7%). In 82 (51.6%) patients the investigators considered the adverse event as possibly related to atomoxetine. Adverse events reported in more than 5% of the patients and rated as possibly related to atomoxetine were: fatigue (N = 42, 26.2%), nausea (N = 22, 13.8%), headache (N = 15, 9.4%), upper abdominal pain (N = 11, 6.9%), decreased appetite (N = 11, 6.9%), dizziness 9 (5.7%) and vomiting 9 (5.7%). There were 8 patients with serious adverse events, which were considered related to atomoxetine in two patients (1 patient with severe vomiting; 1 patient with abdominal pain, dissociation, disturbance in attention, dizziness, fatigue and peripheral vasoconstriction with dark, marbled skin).

Treatment-emergent adverse events led to discontinuation in 7 (4.4%) patients: alopecia, decreased appetite, drug abuse (acute intoxication with unknown medication plus alcohol, hospitalized at emergency unit, then at inpatient adolescent psychiatry ward) fatigue, vasoconstriction (patient above, with additional symptoms/events, hospitalized for diagnostic process), vertigo and vomiting in 1 (0.6%) patient each. Except for fatigue and drug abuse, all these adverse events were rated as possibly related to treatment.

Mean laboratory parameters, including liver function tests, were found within normal ranges with only minor fluctuations over the course of the study (observed cases; ALT: BL 19 ± 8 U/L; wk 8, 18 ± 9; wk 24, 17 ± 7. AST: BL 28.0 ± 6 U/L; wk 8, 28 ± 9, week 24, 26 ± 5). For vital signs, increases in systolic and diastolic blood pressure (SBP, DBP) and heart rate were observed as: SBP: BL, 112.6 ± 13.9 mmHg; wk 8, 115.4 ± 12.9, wk 24, 117.5 ± 12.1. DBP: BL, 70.3 ± 10.0 mmHg; wk 8, 72.8 ± 8.8; wk 24, 73.8 ± 9.2. Heart rate: BL, 77.9 ± 10.7 bpm; wk 8, 87.1 ± 13.6; week 24, 84.8 ± 12.0.

## Discussion

To our knowledge, this is the largest single study focusing on adolescent ADHD patients treated with atomoxetine [18,19]: 159 adolescent patients with ADHD according to DSM-IV-TR were included in this open-label trial with atomoxetine in Germany. The retention rate over the 24-week course of the study was 69.8%, a result closely resembling the 6-month retention rate of 64.9% reported by Perwien et al. from an atomoxetine study in N = 912 patients aged 6–17 years [30]. Patients in this trial were treated with atomoxetine at a mean dose very close to the target dose recommended in the summary of product characteristics (SPC). The rates of psychiatric comorbidities were low compared to studies performed in children. And, interestingly, there was a more than 5-year time window between first occurrence of symptoms and professional diagnosis, approximately 2 years longer than observed, e. g., in the child study conducted in parallel [37].

As the primary objective, we investigated possible differences in perceptions of ADHD-related difficulties by patients, parents, and physicians using the recently devised GIPD instrument [36]. In evaluating the respective findings it has to be pointed out that GIPD instrument and CGI-S scale – although similar in the wording and format of a 7-point Likert scale – should not be seen as fully analogue. While the investigator assesses CGI-S in comparison to the group of patients with the same disorder [cf. instructions; [23,24]], GIPD ratings by the three different raters are to be done without that reference. Thus, GIPD baseline mean values and changes over time may not be understood and interpreted in the same way. When considering discriminant validity in the recent validation paper [36] (including data from this sample), a monotone but non-linear increase was observed when relating GIPD total scores to the CGI-S levels. In comparison to physician and parent ratings, patient-rated GIPD total scores increased to a much lower degree with increasing CGI-S scores.

In this study with adolescent ADHD patients, the degree of the ADHD-related difficulties was rated statistically significantly lower by the patients than by their parents or their physicians. In contrast, the difference between the parent and physician GIPD ratings was small. With reference to the methodological approach chosen in this trial, it appeared that these patients subjectively perceived the degree of their ADHD-related difficulties as lower than their parents or physicians did. These findings were consistent with the parallel open-label study in children with ADHD [37], as well as with the recent double-blind study in children and adolescents [10].

Interestingly, the baseline self-reports of adolescent ADHD patients with the combined subtype vs. the predominantly inattentive subtype did not differ as to the severity of perceived ADHD-related difficulties. In contrast, parents and physicians rated the difficulties of the patients with the combined type as slightly more severe (n. s.) than those of the patients with the predominantly inattentive type, potentially corresponding to the clinical observation of third-party raters' assessing the impact of inattention as less burdensome.

The differences between the three perspectives persisted over the entire observation period. At the end of the acute treatment period (week 8), GIPD Total scores were statistically significantly reduced for all three rater groups (mean change from baseline: patients -2.9, -23.2%, parents -5.9, -34.3%, physicians -7.8, -41.5%), This level of improvement persisted until week 24. In general, these findings also held for the single-item GIPD evening and morning assessments. This statistically significant and consistent decline in ADHD-related difficulties during atomoxetine treatment may cautiously be interpreted to reflect an improvement in patient overall impairment, social functioning, and QoL in this adolescent patient population. Again, findings were highly consistent with the parallel study in children [37]. In addition, a secondary analysis of pooled data from both studies showed improvements in emotional well-being as measured by ten specific items from the Pediatric Adverse Event Rating Scale (PAERS) [41,42]. To evaluate the clinical significance and daily-life impact of these GIPD changes, further investigation will be needed, under controlled conditions, potentially studying additional outcome parameters, populations, and/or comparator compounds.

Previous studies that used more specific instruments to assess QoL in children and adolescents with ADHD [8,10,14,28-30,43], such as the Child Health Questionnaire (CHQ) [25] or the Child Health Illness Profile (CHIP) [10,26], had reported similar findings. For example, Klassen et al. [32] and Riley et al. [6] observed health-related QoL scores of young ADHD patients of up to two standard deviations below population norms reflecting marked impairments. Further, a recent double-blind, placebo controlled 12-week study of atomoxetine treatment in 151 children and adolescents aged 6–15 years also found that HRQoL, as assessed by parents and patients using the CHIP instrument, improved during atomoxetine treatment [10]. Patient-rated QoL impairment was less severe than parent-rated impairment, and correlation between QoL ratings (CHIP) and clinical disease severity (ADHD-RS) was lower for patients' than for parents' [10].

As summarized by Steele et al. [45], studies with sustained-release formulations of stimulants also found ADHD symptom reduction and improvement in functional outcomes, e.g. in measures of social play and parental stress, fewer accidents and injuries, better driving performance and fewer general practitioner visits.

Cohen's Kappa coefficients varied considerably across the three rater groups and the course of the study, with strongest (moderate) agreements between physician and parent assessments, and significant improvements of patient/physician agreements over time. Study design features, with choosing a nearly naturalistic sequence of applying instruments and using information as in an office-setting, may have impacted on this outcome.

With respect to the secondary objectives of this study, mean ADHD-RS Total scores decreased statistically significantly over time, with -15.6 (-54.9%) mean change, from a relatively low baseline (28.4) to week 8 (12.9) in this adolescent population. Wilens et al. [18] reported related findings from a meta-analysis of six placebo-controlled acute studies) with an ADHD-RS baseline score of 36.3, and -38.5% mean change to endpoint. The high rate of 45.9% adolescent patients with predominantly inattentive ADHD-subtype in our study sample may have contributed to the low mean baseline score and the high relative change in ADHD symptoms for the entire sample. Very similar results as here were derived from another meta-analysis by Wilens et al. based on 13 long-term atomoxetine studies with adolescent ADHD patients [19]: ADHD-RS baseline score 34.7, and -58.2% mean change to endpoint. Finally, an analysis from seven placebo-controlled trials by Adler et al. showed significantly larger treatment effects in adolescents vs. adults (baseline ADHD-RS score 37.3) [20].

The percentage of ADHD-RS mean change for adolescents reported here was generally in line with several placebo-controlled trials – involving both children and adolescents – which showed efficacy with atomoxetine treatment [8,13-15]. In these double-blind studies (1.2 mg/kg/d arm from Michelson et al., 2001 [8]), e.g., mean ages ranged from 9.7 to 11.5 years, baseline mean ADHD-RS scores from 37.6 to 42.1, and % mean changes from -34.0 to -39.7%. The respective values from our open-label study performed in children (N = 262; 6 to 11 years) were: 9.3 years, 35.2, and -38.9% [37].The current findings support the notion that treatment with atomoxetine is effective, since symptom reductions in these open-label studies were similar to those in randomized, placebo-controlled trials, with certain differences for the child and adolescent populations. They may also suggest that open-label study results do not necessarily reflect observer bias towards higher effectiveness compared to double-blind placebo-controlled trials.

Based on ADHD-RS assessments, effectiveness was shown both for the combined type and the predominantly inattentive type of ADHD. This finding could be anticipated since atomoxetine has been known to improve both hyperactive/impulsive and inattentive symptoms of ADHD [8,13,15,44]. The ADHD-RS total score at baseline was significantly higher in patients with combined type ADHD than in patients with predominantly inattentive type ADHD, as to be expected. Furthermore, in this study, the baseline mean ADHD-RS total score in girls was observed to be below the ratings for boys. Some reports indicate that core ADHD symptoms observed in girls are similar to those seen in boys with ADHD [16,46]. But there are also findings that girls with ADHD appear to be less hyperactive-impulsive and thus possibly less impaired than boys [47].

Mean CGI-S scores also significantly improved over time [cf. [8,13,15]], this applied both to the group with combined type ADHD and to the group with predominantly inattentive type ADHD. CGI-S scores in patients with combined type ADHD were reported above that in patients with predominantly inattentive type ADHD. Together with the results from the ADHD-RS these findings seem to support the notion that the subtype of ADHD may to some extent be a reflection of overall ADHD severity. This has also been suggested for comorbid oppositional defiant disorder (ODD) which may also reflect overall ADHD severity [44].

In general, the pattern of treatment-emergent adverse events in this study appeared to be in line with that reported in randomized, placebo-controlled trials in children [13,15,16] or children and adolescents [8], respectively. Compared to these studies, only the rates of fatigue and nausea were relatively high. This may reflect a different pattern of physical complaints and adverse events in an adolescent population, or be due to the open-label design of this study. Changes in laboratory parameters and vital signs were not considered clinically relevant.

The reported data from this study should be interpreted cautiously in the context of its methodological limitations: The open-label study design in general is prone to rater bias, although symptom improvements over time were corresponding to results from double-blind placebo-controlled trials. And, as often observed when using several instruments, there will be overlap in constructs measured by the various scales [9]. The sequence of applying the scales and instruments in this study may also have influenced the investigator GIPD ratings and, thus, the Cohen's Kappa values indicating moderate parent/physician agreement. In the absence of comparator groups (e.g., placebo, somatic or other mental disorders) findings remain open to interpretation. In retrospect, it has also to be acknowledged that our study design and the data as collected did not allow to explore and investigate further the clinical and practical relevance of the differences observed between raters. Issues related to actual impairment and QoL as well as potential impact for differential approaches in psycho-education and treatment of ADHD, both in adolescents and parents, certainly appear to be of great importance. Further, reporting of adverse events may to some degree depend on the timing of the study with respect to the life cycle of a compound; e.g., physicians' experience and perspectives, and thus reporting rates may change with several years of using a medication after market introduction [48,49].

In summary, adolescent patients rated their ADHD-related difficulties, which can be considered to reflect overall impairment, social functioning, and QoL, as lower than either parents or physicians did. All three rater groups reported clear reductions in severity of these perceived difficulties over time, for total, evening, and morning assessments. The different views, the patient perspective on daily difficulties in particular, may provide important additional information when evaluating effectiveness of a treatment [cf [36]] and adjusting components of therapy individually. ADHD core symptoms also improved over the course of treatment, and atomoxetine was generally well tolerated.

The reported differences in overall GIPD ratings and their statistical relevance may guide clinicians and researchers to collect more detailed differential data on actual impairment and quality of life from both adolescent ADHD patients and their parents, if applying more elaborate techniques and measures, both in clinical work and in future studies focussing on HRQoL. Additionally, comparative research is needed on the impact of various ADHD medications, focusing not only on the core symptoms of ADHD but as well on the health-related quality of life and functional outcomes of both patients and families, considering age and gender effects across the life-span.

## Competing interests

RWD is a former employee of Lilly Deutschland and now holds an Eli Lilly Endowed Chair of Pediatric Psychopharmacology. PMW, AS, AM, and ML are full-time employees of Lilly Deutschland, RWD and PMW own Eli Lilly & Co. stock. GL has received research grants and speaker honoraria from Eli Lilly & Co. and is member of a Lilly Advisory Board.

## Authors' contributions

RWD, PMW, ML and AS developed the clinical trial. AS developed the analyses reported in this manuscript. All authors participated in development of the GIPD scale and the interpretation of data. RWD, AS and AM drafted the manuscript, PMW, ML, KS and GL revised it critically for important intellectual content. All authors read and approved the final manuscript.
